# Retroperitoneal Necrotizing Fasciitis Masquerading as Perianal Abscess – Rare and Perilous

**DOI:** 10.7759/cureus.982

**Published:** 2017-01-17

**Authors:** Anandhi Amaranathan, Ashok Kumar Sahoo, Deepak Barathi, Gomathi Shankar, Sarath Chandra Sistla

**Affiliations:** 1 General Surgery, Jawaharlal Institute of Postgraduate Medical Education and Research (JIPMER), Puducherry, India.; 2 Radiology, Jawaharlal Institute of Postgraduate Medical Education and Research (JIPMER), Puducherry, India.

**Keywords:** necrotising fasciitis, retroperitoneal, surgical care

## Abstract

Necrotizing fasciitis is one of the uncommon presentations of a rapidly spreading subcutaneous tissue infection. Although the actual cause is unclear in many cases, most of them are due to the rapid proliferation of microorganisms. Retroperitoneal necrotizing fasciitis is extremely rare. It is a potentially lethal infection that requires immediate and aggressive surgical care. Early diagnosis is the key to a better prognosis. The possibility of retroperitoneal necrotizing fasciitis should be suspected in patients with symptoms of sepsis that are disproportionate to clinical findings. The rapid deterioration of the patient also gives a clue towards the diagnosis.

We report a 35-year-old male with perianal abscess who had been progressed to retroperitoneal necrotizing fasciitis. The patient was managed successfully with aggressive debridement and drainage after laparotomy. Appropriate antibiotics were used to combat the sepsis. The patient recovered well at follow up, three months after discharge.

Another patient, a 45-year-old male with a retroperitoneal abscess, progressed to retroperitoneal necrotizing fasciitis, and extra peritoneal drainage and debridement was done. Antibiotics depending upon the culture and sensitivity were used to control sepsis. But the patient succumbed to death 45 days after surgery due to uncontrolled sepsis.

Necrotizing fasciitis of any anatomical site needs aggressive surgical care with early intervention. But retroperitoneal necrotizing fasciitis needs an extra effort for diagnosis. After diagnosis, it needs timely surgical intervention and appropriate antibiotic therapy for the recovery of the patients.

## Introduction

Necrotizing fasciitis (NF) is an uncommon but potentially life-threatening infection which involves the subcutaneous tissue and fascia. The more popular name for it is flesh-eating disease. The incidence of necrotizing fasciitis is 500–1500 cases per year with a mortality rate of 24%–34% [1]. It is not so easy to differentiate NF from cellulitis and other superficial skin infections at the initial stage. In 1871, Jones first described necrotizing skin infections as “hospital gangrene” [2]. Wilson, in the 1950s, coined the term “necrotizing fasciitis”, which was defined as necrosis of the fascia and subcutaneous tissue with relative sparing of the underlying muscle [2]. The characteristic features of necrotizing fasciitis are rapid tissue damage, systemic toxicity, and if there is a delay in treatment, it causes severe morbidity and mortality. In the majority of cases, the etiology is polymicrobial, with the most common microorganism being the streptococcus species [3]. Other reported organisms are Klebsiella pneumonia, Escherichia coli, Streptococcus pyogenes, fecalis and pneumonia, Staphylococcus aureus, Bacteroides fragilis, and Clostridia septicum [2]. Various imaging modalities like ultrasound (US), computed tomography (CT), and magnetic resonance imaging (MRI) have vital roles in the diagnosis of NF. Characteristic imaging findings on CT have been described and include thickening of the fascial planes, soft-tissue gas, and abscess formation [4]. Early surgical exploration and aggressive debridement along with appropriate antibiotic therapy are the principal modes of treatment.

Necrotizing fasciitis involving the retroperitoneum is very uncommon. There are no specific signs and symptoms of retroperitoneal necrotizing fasciitis. The various modes of presentation are indolent fevers and chills; weight loss; abdominal, flank, groin, or back pain; malaise; and anorexia. Due to the nonspecific clinical presentations, diagnosis is usually delayed, often for months, or is confused with other clinical entities. Sometimes, patients may present with the rapid onset of septic shock or other complications after the perforation and release of bacterial contents into various body cavities like the peritoneum, thorax, meninges, and thigh.

Informed consent was obtained from all the patients for this study.

## Case presentation

### Case report 1

A 35-year-old male presented to causality with chief complaints of swelling in the perianal region and fever for four days. The swelling was insidious in onset and ruptured spontaneously with the discharge of foul smelling pus. The fever was of high grade and intermittent with chills and rigor. He complained of mild discomfort on the left side back. History of decreased urine output was there for one day along with loss of appetite. He had complaints of constipation for four days. He was not suffering from chronic illnesses like diabetes mellitus, hypertension, or tuberculosis. He was neither a smoker nor an alcoholic.

On general examination, the patient was conscious, oriented, and afebrile. The patient had mild dehydration and tachypnea at rest. Vitals signs and systemic examination of the cardiac, respiratory, and nervous systems were normal. The abdominal examination revealed distension which was diffuse with tenderness present over the left lumbar and inguinal region, but with normal bowel sound and no peritoneal signs. On local examination of the perianal region, 3X4 cms ulcer in the right perianal region with active pus discharge wasfound. Tenderness was present in the lateral walls during per rectal examination. The patient was posted for incision and drainage under anesthesia and drained of 15 ml pus.

Laboratory investigations showed leukocytosis (14,410/ml) with neutrophilia. Electrolytes and renal parameters were normal. Postoperatively, the patient had persistent pus discharge from the perianal wound.

On the second postoperative day, he complained of lower abdominal pain and distension. The ultrasonography revealed a 7X6X3 cms collection from the left lumbar region extending to the left pelvis in the retroperitoneal plane with moving echoes. Bowel loops were normal. Pigtail was placed in the collection and around 200 ml pus was drained. 

**Figure 1:**
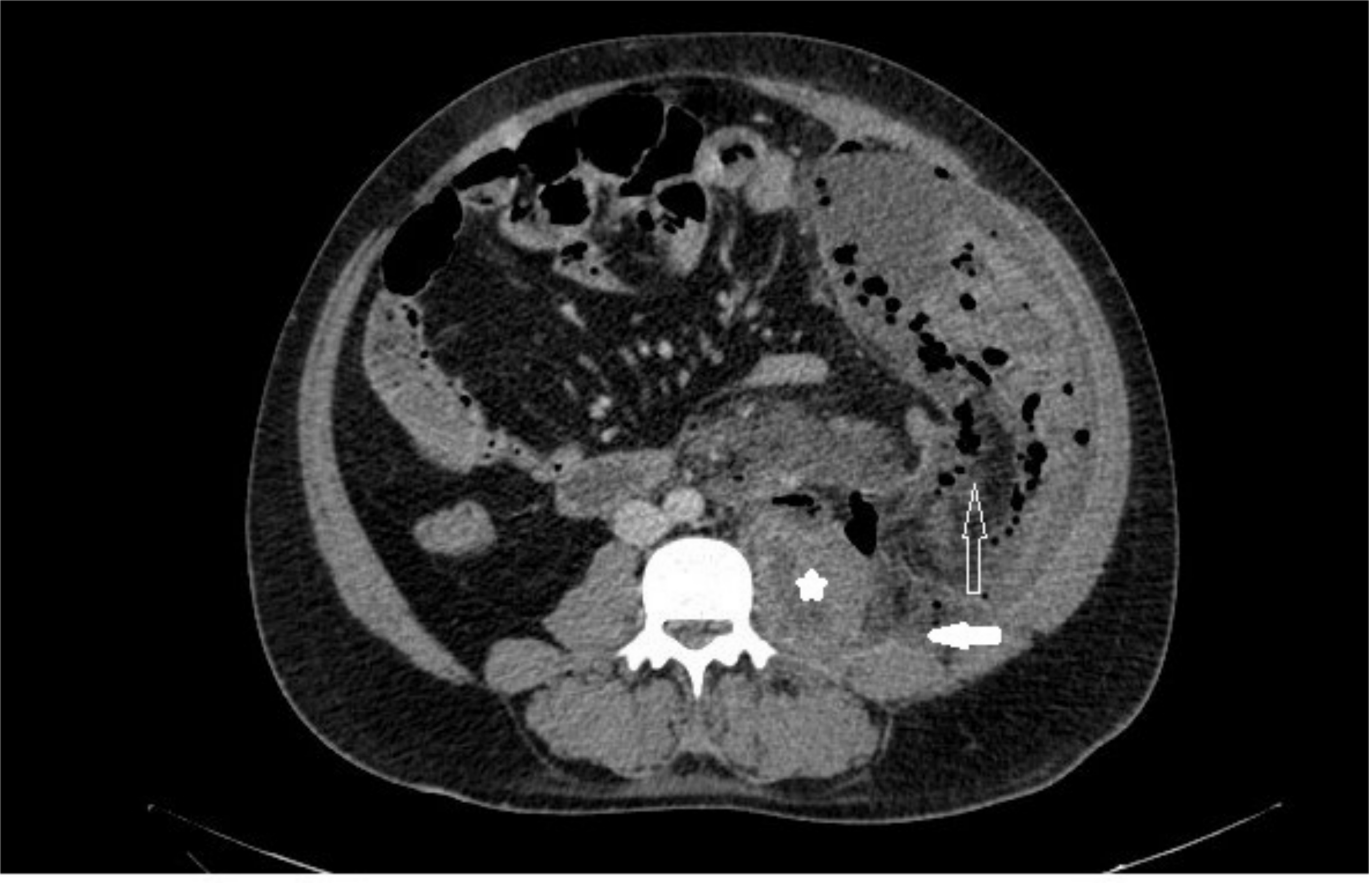
Axial section of CECT abdomen showing enlarged left psoas muscle with ill-defined hypodense areas (star) and retroperitoneal fat stranding (solid arrow). Fluid collection with multiple air pockets are seen in the left posterior preperitoneal space (hollow arrow).

**Figure 2:**
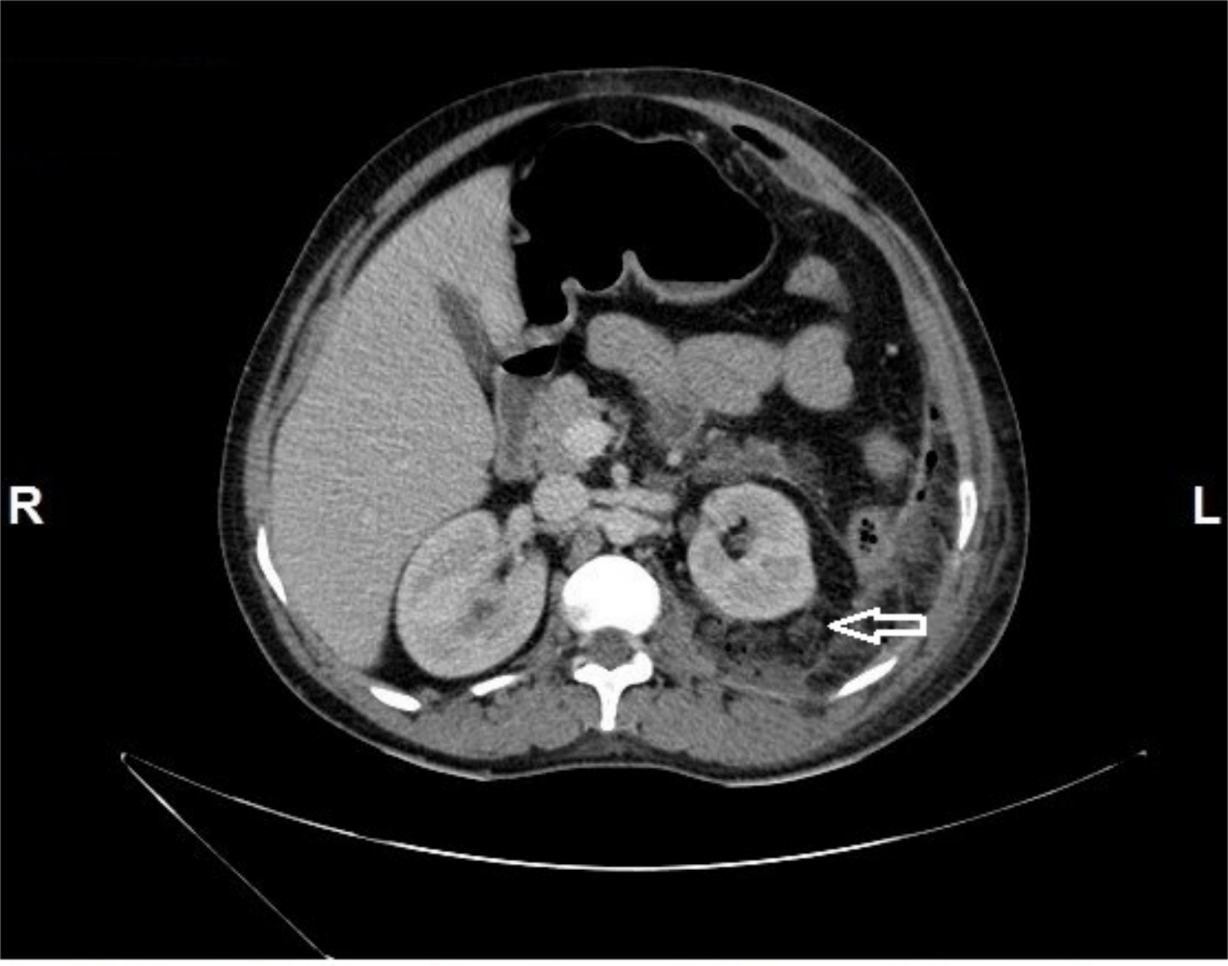
Axial section of CECT abdomen showing stranding of left perinephric fat with multiple air pockets (arrow).

A contrast-enhanced computed tomography (CECT) scan (Figure 1 and 2) of the abdomen and pelvis was performed, demonstrating a heterogeneous collection of size 21X9 cms with multiple air pockets in the left pro-peritoneal layer in the left lumbar and iliac fossa region extending along the left iliopsoas, retroperitoneal, pelvic wall, and ischiorectal fossa. 

**Figure 3:**
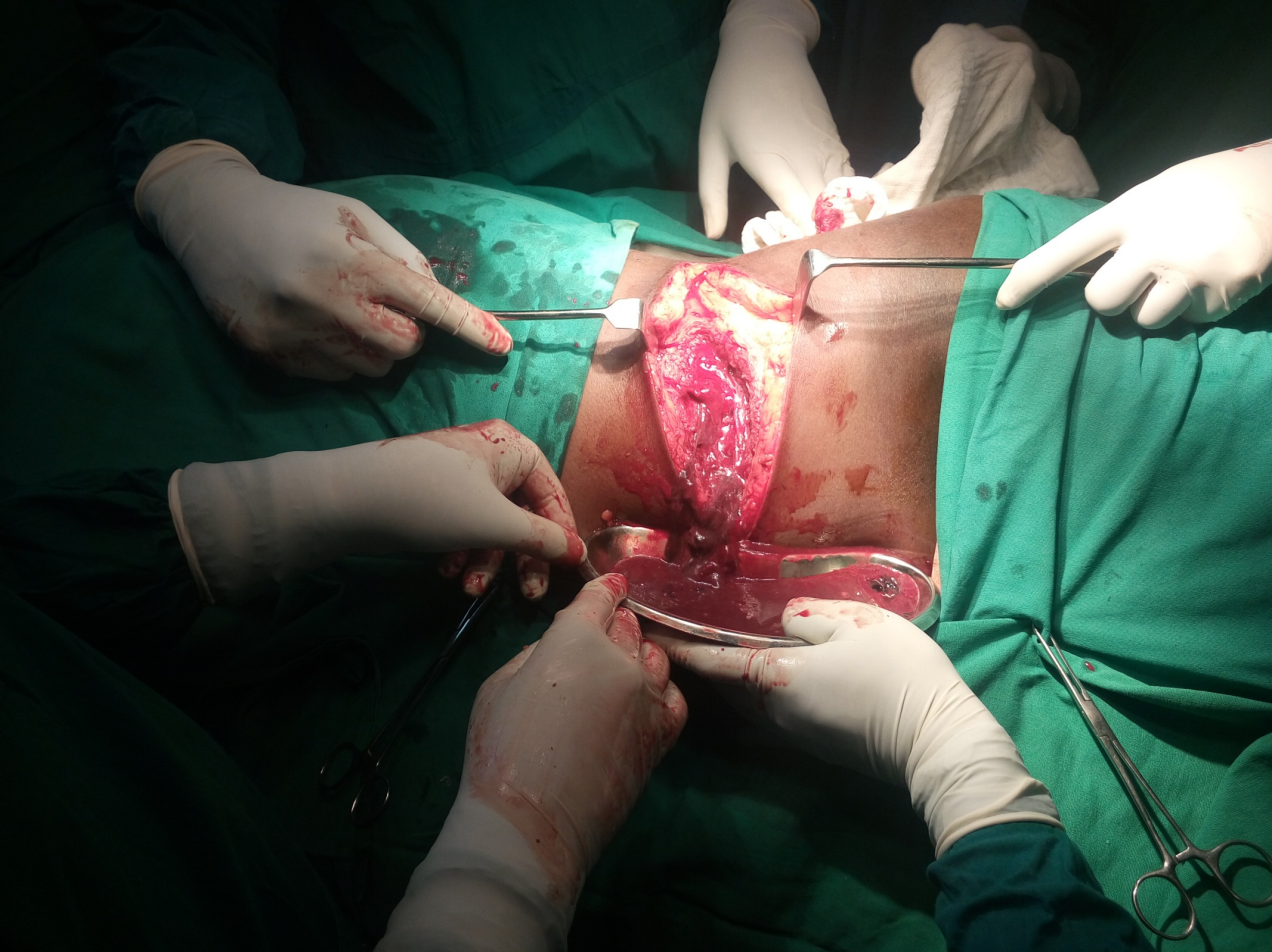
Intraoperative picture showing left loin incision and drainage of pus

 In view of the deteriorating condition of the patient, he had been posted for incision and drainage under anesthesia following the fourth day of pigtail insertion. Since the necrotizing fasciitis was retroperitoneal, we used a left loin incision and drained around 400 ml pus with extensive debridement of necrotic tissue. The peritoneal cavity was not breached (Figure 3).

The abscess cavity extended up to the inframammary line anterosuperiorly, up to inguinal region antero inferiorly, postero superiorly till angle of the scapula, and posteroinferiorly till the iliac crest. The cavity had not crossed the midline. The left kidney and duodenum were normal. A left flank drain was placed. Postoperatively, thrice daily cleaning and dressing of wound was done. Antibiotics were given according to culture and sensitivity of the pus. Cultures had grown Escherichia coli, Staphylococcus aureus, Acinetobacter lwoffii, and Pseudomonas aerogenosa. Pigtail was removed on postoperative day five followed by the left flank drain on the eleventh day. Wound care was done by continuous irrigation and sitz bath for the perianal wound. The loin wound was closed by secondary suturing on the 20th day after surgery, and the patient was discharged on the 25th day. The patient is doing well in the third month of his follow up.

### Case report 2

A 45-year-old male laborer presented to casualty with chief complaints of abdominal pain for one month and abdominal distension and vomiting for two weeks. The abdominal pain was a dull aching type, intermittent, and diffuse, but increased in intensity since one day. He had a history of diffuse abdominal distension. He had the vomiting for two weeks which was of multiple episodes and containing food particles with no blood. He had bilateral lower limb swelling for one week. The patient had denied of any fever, chest pain, melena, a decrease in urine output, and any co-morbidities. He was a chronic alcoholic for 15 years.

General examination revealed that the patient was conscious, oriented, and afebrile but with mild dehydration and bilateral lower limb edema. Vitals were pulse rate – 100/min, blood pressure – 70/60 mm of Hg, spO2 – 98%. Cardiovascular, respiratory, and central nervous systems revealed no abnormality. On abdominal examination, it was soft but diffusely distended with hepatosplenomegaly. There was tenderness over the right hypochondriac and epigastric region with the presence of shifting dullness. Per rectal examination was normal. The patient was resuscitated with appropriate fluid till hemodynamically stable.

The ultrasound of abdomen revealed acute pancreatitis with parenchymal liver disease, moderate ascites, mild right pleural effusion, splenomegaly, and bilateral medical renal disease. The patient was posted for emergency laparotomy in view of the deterioration of the general condition and ongoing sepsis.

**Figure 4:**
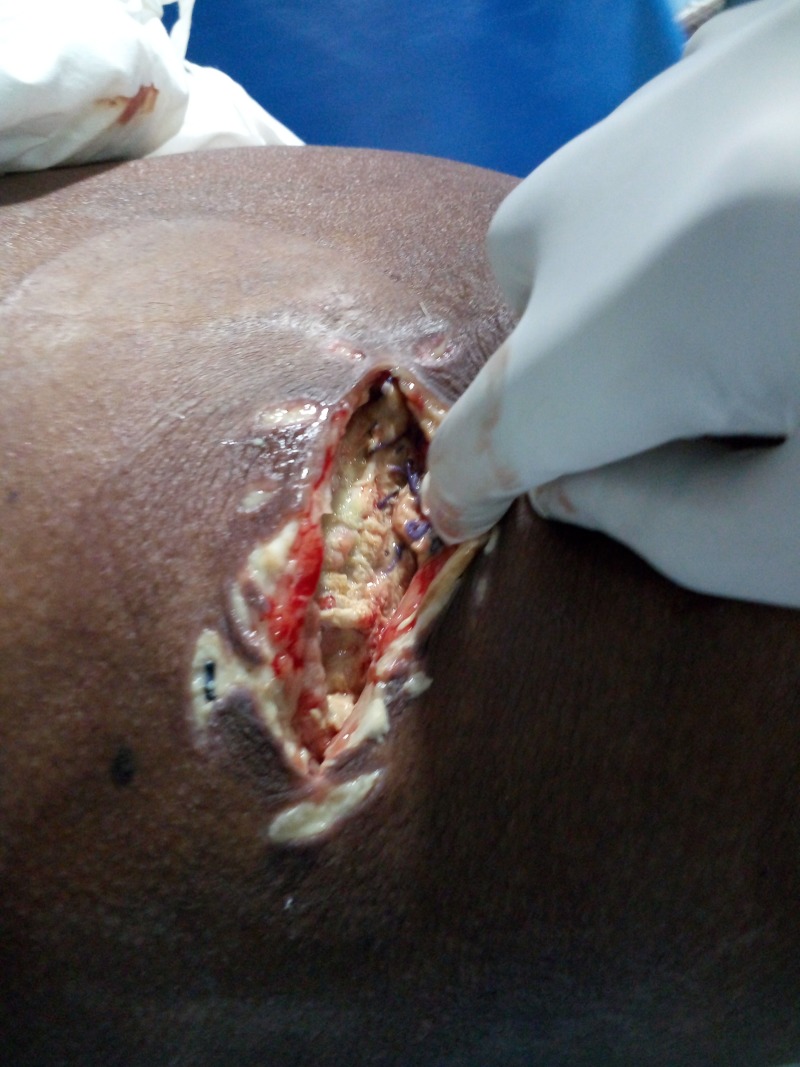
Intraoperative picture showing incision and drainage

Intraoperative findings (Figure 4) revealed 300 ml of pus in the retroperitoneum posterior to the ascending colon, extending from the subhepatic region to inguinal region. Thorough lavage was given followed by bilateral drain placement in the left pelvis and subhepatic region.

The patient was started on appropriate antibiotics according to the culture and sensitivity of pus and blood. Pus and blood culture had grown Klebsiella pneumonia and Escherichia coli.

**Figure 5:**
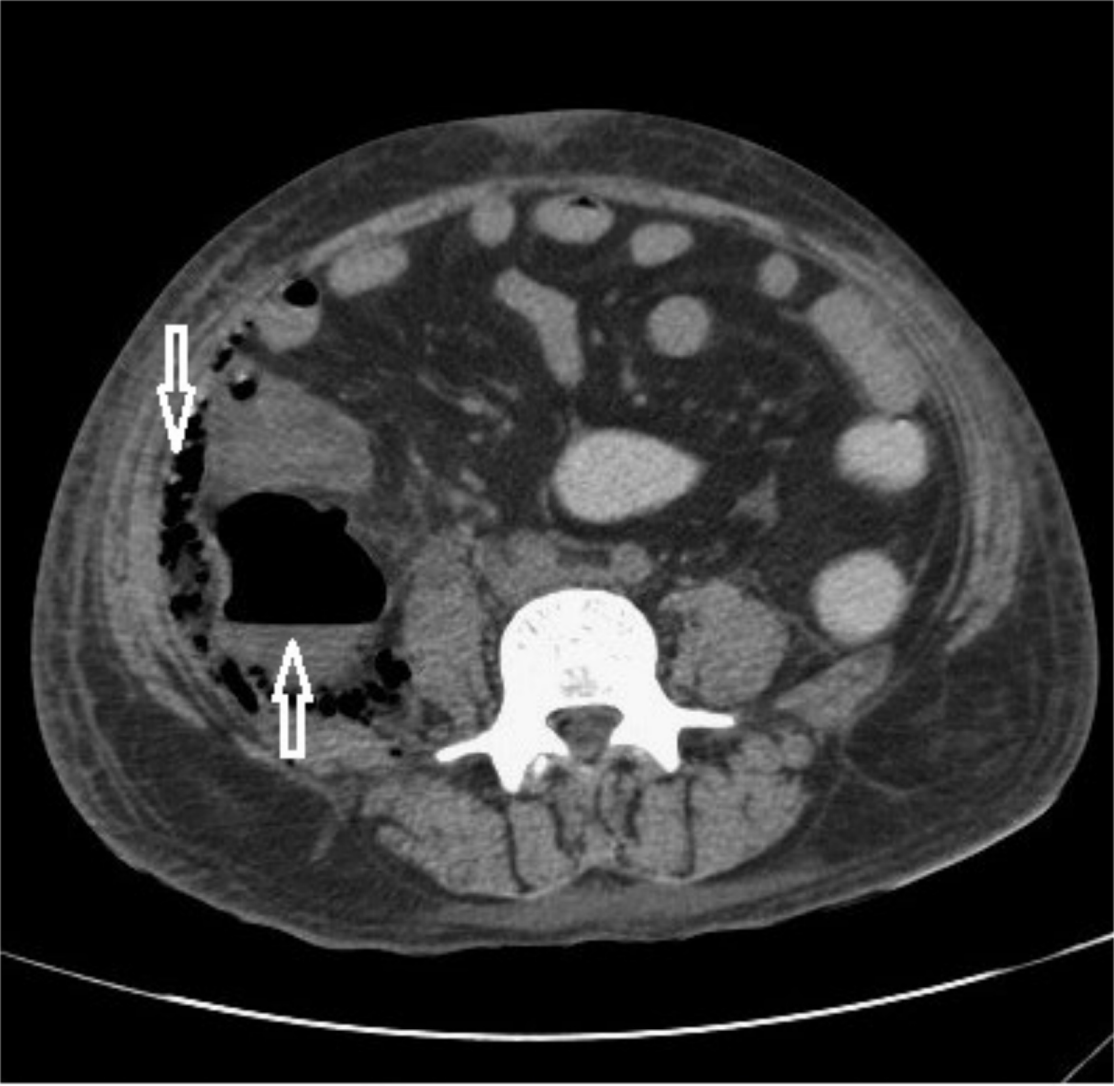
Axial section of CECT abdomen shows collection with air fluid level in the retrocaecal region (upward arrow) and multiple air pockets in the right posterior pararenal space extending along the preperitoneal space (downward arrow).

**Figure 6:**
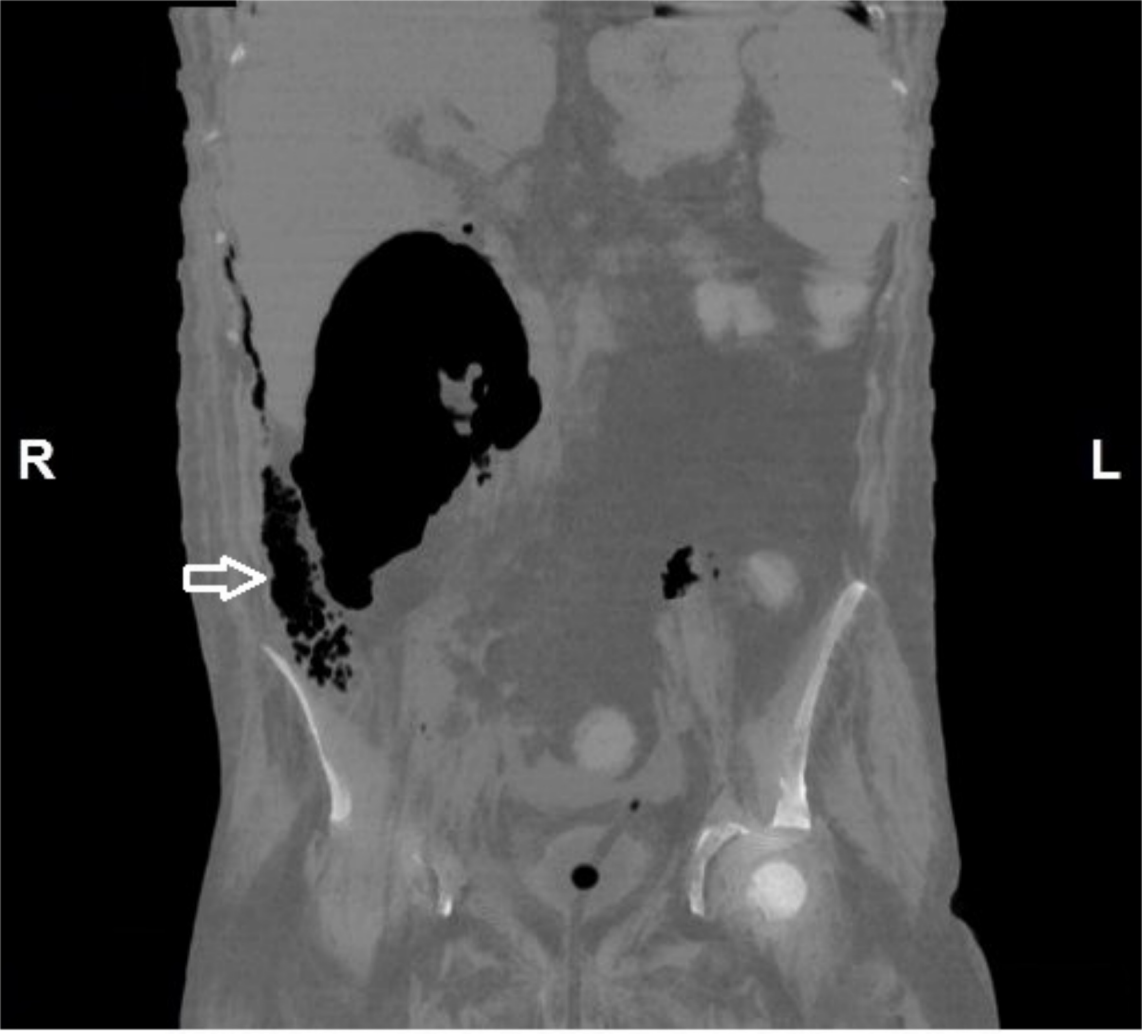
Coronal section of abdomen shows the extent of large retrocaecal air collection and also multiple air specks in the preperitoneal plane (arrow).

The patient was shifted to the intensive care unit (ICU) and was on the ventilator for ten days postoperatively. So tracheostomy was done as the patient could not wean off the ventilator. In view of the deterioration of the condition of the patient and ongoing sepsis, CECT (Figure 5 and 6) was done on the 32nd postoperative day.

It showed residual fluid in the retroperitoneum around the ascending colon and in the right perinephric region. There were irregular cavitating nodules in the bilateral lung parenchyma (possibility of septic emboli), hepatosplenomegaly, massive ascites, and moderate left pleural effusion.

According to blood culture and tracheal aspirate culture, antibiotics were given. Laboratory values revealed leukocytosis (34,000 to 64,000) with neutrophilia and thrombocytopenia. Electrolytes and renal parameters were normal initially but became deranged as sepsis progressed. The patient could not recover and succumbed to death on the 47th postoperative day.

## Discussion

Necrotizing fasciitis is a serious soft tissue infection that causes the secondary necrosis of soft tissues. It is of two types depending upon the etiology. Type I is of polymicrobial origin, while type II is due to monomicrobial infection; the latter constitutes the majority of cases (80%) [5]. The cases in this report were classified as type II. The most important principles of management of this life-threatening infection are early recognition, appropriate broad-spectrum antibiotic treat­ment, and aggressive surgical debridement. Due to the masquerading features of necrotizing fasciitis with cellulitis, it is very difficult to diagnose it at its earliest stage. Necrotizing fasciitis of the anterior abdominal wall or the perineum usually presents as erythema and induration of the overlying skin.

The earlier exploration of retroperitoneal necrotizing fasciitis is delayed due to its nonspecific clinical manifestation. The main hindrance to curative debridement in the retroperitoneum is its complicated anatomy. Moreover, these patients usually have severe comorbid conditions like immunosuppression or advanced neoplastic disease. Due to all these factors, the mortality rate in retroperitoneal necrotizing fasciitis is high. Woodburn [6] reported five patients with retroperitoneal necrotizing fasciitis, all of whom died. Three of them had colon cancer while one had a diverticular abscess and another had T-cell lymphoma. A few cases of survival have been reported in cases of retroperitoneal fasciitis [7-8]. A 4-year-old girl who suffered from the infection following pyelonephritis was treated with debridement and antibiotics [2]. In a South African study, eight cases survived from the affected ten cases [9]. They harbored the condition secondary to caesarean section, perineal sepsis, intrauterine death, intrauterine instrumentation, and trauma.

The usual mode of presentation of perianal abscesses is severe perianal pain, fever, and sometimes purulent discharge. The treatment of choice is complete and appropriate drainage. The most feared complication after the drainage of an anorectal abscess is a fistula in ano. The development of a very severe complication like necrotizing fasciitis is very uncommon but may spread either to the peritoneal or retroperitoneal spaces. Our patient had a perianal abscess which progressed to retroperitoneal necrotizing fasciitis.

Zaveri, et al. [10] reported a young male without any comorbid condition, who presented with a retroperitoneal extension of a perianal abscess after initial drainage. He developed continuing sepsis, abdominal distension, and pain. The drainage was done through a lumbar incision based on the X-ray finding of gas in the retroperitoneum. In spite of that, the patient did not survive. The presentation of our first case is similar to that described by Zaveri, et al. Several factors could have helped contribute to the positive outcome for our patient. These include our patient’s age and the absence of any comorbidities; urgent incision and drainage were carried out through a wide lumbar incision without breaching the peritoneal cavity along with the extensive debridement of necrotizing tissues. Appropriate antibiotic treatment, good nursing care, and proper metabolic and nutritional support would have contributed to the smooth recovery of the patient.

We report another case that developed extensive necrotizing fasciitis of the retroperitoneum. The diagnosis was confirmed intraoperatively with subsequent debridement. Most of the cases of retroperitoneal necrotizing fasciitis in the literature had a known primary source of infections like chronic pyelonephritis, diverticulitis, perianal abscess, colonic cancer, perforation, urinary extravasations, and post hemorrhoidectomy [8]. As with our patient, there have been cases of retroperitoneal necrotizing fasciitis reported without an obvious source of infection [8]. Our patient revealed a history of abdominal pain for one month with an aggravation of pain for one day. We hypothesize that the patient developed a perforation which manifested late in a subacute manner. While retroperitoneal necrotizing fasciitis is more common in immunocompromised patients [6], our patient had no history or clinical findings suggestive of diabetes mellitus, chronic renal failure, human immunodeficiency virus, or any other obvious immunosuppressed state. The survival rate in retroperitoneal necrotizing fasciitis is very less [7-8].

The ultimate treatment for necrotizing fasciitis is early radical debridement of the affected necrotic tissues [6] with appropriate broad spectrum antibiotic therapy to cover a wide range of potential pathogens. Retroperitoneal involvement, however, makes early curative debridement unfeasible. In the beginning, it affects the pericolic or perirectal sepsis followed by spreading along retroperitoneal tissue planes. The resultant vascular thrombosis causes subsequent ischaemic and infective changes in the overlying skin and subcutaneous tissues, giving the characteristic appearance of necrotizing fasciitis. The late manifestation of these changes in the overlying cutaneous tissues results in delayed surgical intervention, by which time curative resection is not feasible. There are no investigations available till date for early diagnosis of retroperitoneal necrotizing fasciitis. The presence of retroperitoneal gas on the plain radiograph is not diagnostic of retroperitoneal necrotizing fasciitis. A CT scan is the best investigation to diagnose the development of retroperitoneal necrotizing fasciitis, but these changes were compatible with an advanced and ultimately fatal presentation. No imaging technique will improve the prognosis for retroperitoneal necrotizing fasciitis unless it is coupled with a high degree of clinical awareness and early surgical intervention. Antibiotic therapy appears to be of secondary importance to achieving adequate surgical excision. The use of antibiotic therapy at an early stage may be beneficial in reducing the amount of tissue destruction.

## Conclusions

In conclusion, even the benign looking perianal abscess, if it not showing improvement even after adequate surgical clearance, should raise suspicion of more extensive involvement of the tissue and retroperitoneal spread. The findings of rapidly evolving cellulitis in the clinical setting and of soft-tissue gas either clinically or on imaging studies should alert the clinician to the possibility of necrotizing fasciitis. Appropriate management should be initiated, including immediate intensive medical treatment, resuscitation, and surgical referral with a view to immediate exploration and debridement under appropriate antibiotic coverage.
